# Hypoxia-Induced Retinal Angiogenesis in Zebrafish as a Model to Study Retinopathy

**DOI:** 10.1371/journal.pone.0002748

**Published:** 2008-07-23

**Authors:** Renhai Cao, Lasse Dahl Ejby Jensen, Iris Söll, Giselbert Hauptmann, Yihai Cao

**Affiliations:** 1 Department of Microbiology, Tumor and Cell Biology, The Karolinska Institute, Stockholm, Sweden; 2 School of Life Sciences, Södertörns University College, Huddinge, Sweden; 3 Department of Biosciences and Nutrition, The Karolinska Institute, Huddinge, Sweden; University of Cincinnati, United States of America

## Abstract

Mechanistic understanding and defining novel therapeutic targets of diabetic retinopathy and age-related macular degeneration (AMD) have been hampered by a lack of appropriate adult animal models. Here we describe a simple and highly reproducible adult *fli*-EGFP transgenic zebrafish model to study retinal angiogenesis. The retinal vasculature in the adult zebrafish is highly organized and hypoxia-induced neovascularization occurs in a predictable area of capillary plexuses. New retinal vessels and vascular sprouts can be accurately measured and quantified. Orally active anti-VEGF agents including sunitinib and ZM323881 effectively block hypoxia-induced retinal neovascularization. Intriguingly, blockage of the Notch signaling pathway by the inhibitor DAPT under hypoxia, results in a high density of arterial sprouting in all optical arteries. The Notch suppression-induced arterial sprouting is dependent on tissue hypoxia. However, in the presence of DAPT substantial endothelial tip cell formation was detected only in optic capillary plexuses under normoxia. These findings suggest that hypoxia shifts the vascular targets of Notch inhibitors. Our findings for the first time show a clinically relevant retinal angiogenesis model in adult zebrafish, which might serve as a platform for studying mechanisms of retinal angiogenesis, for defining novel therapeutic targets, and for screening of novel antiangiogenic drugs.

## Introduction

Pathological angiogenesis in the retina is the leading cause of human blindness resulting from diabetic retinopathy, age-related macular degeneration (AMD), and retinopathy of prematurity (ROP). Despite of significant advances in medical care, diabetic retinopathy, AMD and ROP continue to remain the leading causes of vision impairment and blindness in adults and infants respectively [1]–[4]. These common ocular disorders are characterized by overgrowth of disorganized, leaky and physiologically non-functional retinal vessels, which lead to vision impairment and blindness [1], [4]. A good adult animal model that recapitulates clinical pathology of retinal angiogenesis does not exist [5], [6]. Thus, there is a great and urgent need to develop clinically relevant retinopathy models in both newborn and adult animals for studying mechanisms of pathological neovascularization in the retina, screening of novel drugs, and validating antiangiogenic therapy.

In humans, the posterior part of the eye is nourished by two vascular networks, the choroidal and retinal vascular systems, which nourish the outer and inner layers of the retina, respectively [4], [5], [7]. During embryonic development, the inner layer of retinal vasculature is absent and nourishment of the retina is accomplished by choroidal and hyaloid vessels [8]. The hyaloid vasculature is a transient vascular network, which is attached to the lens and undergoes progressive regression as the retinal vasculature develops and matures. In ROP, the leading cause of blindness in infants, high O_2_ perfusion leads to termination of hyaloid vessel regression and the formation of premature retinal vasculature [4], [9]. A rodent model has been developed to imitate human ROP in which newborn mice are exposed to hyperoxia [10]. Upon return to normoxia, the infant retina is relatively hypoxic leading to pathological angiogenesis. However, this model does not accurately reproduce human ROP.

Many lines of evidence support the idea that alteration of O_2_ levels is the key driving force of initiating pathological neovascularization. These include: 1) Pathological retinal angiogenesis occurs in association with retinal ischemia in several diseases [11], [12]; 2) Development of retinal vasculature in embryos is determined by O_2_ gradients [4], [13]; 3) Hypoxia-inducible VEGF expression levels are spatiotemporally coupled to retinal neovascularization [4], [9], [13], [14]; and 4) Anti-VEGF agents show remarkable therapeutic efficacy in both animal and human ophthalmologic disorders characterized by retinal neovascularization [4], [9], [13]–[21]. These findings point to hypoxia-induced VEGF as the key angiogenic molecule responsible for retinal neovascularization. Indeed, the newly formed retinal vessels in AMD, diabetic retinopathy and ROP share unique features with VEGF-induced vascular networks. For example, pathological retinal vessels are premature, highly disorganized, and leaky. In fact, AMD, diabetic retinopathy and ROP all manifest severe retinal edema, leading to impairment of vision [5].

High VEGF levels in ischemic retinas might reflect a compensatory mechanism by which VEGF intends to improve O_2_ delivery during retinal hypoxia. However, the VEGF-induced vasculature consists of disorganized and leaky primitive vascular networks leading to retinal edema and impairment of vision [22]–[27]. Thus, VEGF has become an attractive key target for development of new drugs against ocular diseases. Not surprisingly, both animal studies and clinical experiences demonstrate that blockage of VEGF signaling is a valid approach for the treatment of AMD although the therapeutic efficacy for diabetic retinopathy and ROP needs further validation in appropriate animal models. VEGF binds to both VEGFR-1 and VEGFR-2 mainly distributed in endothelial cells and it is VEGFR-2 that mediates active angiogenic and vascular permeability functions [28]–[32]. Thus, VEGFR-2 has become an attractive therapeutic target for treatments of several common human diseases including cancer and AMD.

The Notch signaling pathway has recently been highlighted in contributing to pathological angiogenesis [33]–[35]. Notch ligand DLL-4 acts as a negative feedback mechanism downstream of the VEGF signaling pathway and regulates endothelial cell differentiation, arterial sprouting, vascular remodeling and patterning, vessel maturation, and vessel stability [36]. Inhibition of the Notch pathway lead to reduced tumor growth, but increased formation of non-functional vasculature [37], [38]. However, while the role of Notch in vascular development has been studied in embryos or infant animals little is known about its function in adult pathological angiogenesis of non-malignant tissues, and particular its relation to tissue hypoxia [39], [40].

The retinal vasculature of adult zebrafish shares some similarities with that of humans. For example, mural cell coating, endothelial cell junctions, and basement membrane composition are similar in humans and zebrafish [13]. Thus, a retinal neovascularization disease model in adult zebrafish would be invaluable for understanding molecular mechanisms of retinal pathology, defining novel therapeutic targets and for drug validation. In this paper, we for the first time, describe such a highly reproducible ocular disease model in adult zebrafish.

## Methods

### Zebrafish strain and maintenance

*Fli*-EGFP-Tg zebrafish were purchased from the ZIRC fish center (Oregon) and maintained in aquaria according to standard procedures on a 10-h dark/14-h light cycle at 28.5°C [43]. Approximately 5–18-month-old adult zebrafish were used for all experiments. Before experimental operations, all zebrafish were anaesthetized with 0.02% tricaine (Sigma). The zebrafish facility and all experimental procedures were approved by the North Stockholm Experimental Animal Ethical Committee.

### Experimental hypoxia

The hypoxia device was engineered to perfuse N_2_ gas directly into the water *via* an air diffuser and the aquarium was virtually sealed to prevent leaking air. The N_2_ gas flow was engaged/disengaged by a valve, which was controlled by an O_2_ regulator (All from Loligo Systems, Denmark). The O_2_ regulator controlled the O_2_ tension via an electrode and the entire hypoxia system was automated to maintain a constant level of O_2_ in the aquaria water. The temperature of the aquaria water remained at 26°C, which was maintained by using a thermostat. Zebrafish were first placed in normoxic water and the O_2_ tension was gradually reduced to adopt the final 10% air saturation (820 ppb) over the course of 48–72 h. Zebrafish was exposed in this hypoxic environment for different time points with a maximal period of 15 days.

### VEGF and Notch blockage

*Fli*-EGFP-Tg zebrafish exposed in the hypoxic condition as described above were also incubated with 0.5 µM sunitinib or 1 µM ZN323881 for 15 days. The final concentration of the vehicle (sodium citrate or DMSO respectively) was less than 1∶40,000. For Notch inhibition, DAPT (N-[N-(3,5-difluorophenacetyl)-L-alanyl]-S-phenylglycine t-butyl ester; Sigma-Aldrich) from a stock of 10 mM in DMSO was added to the aquaria water to achieve a final concentration of 10 µM and zebrafish were incubated for 5 days and under hypoxia or 6 days under normoxia. During this period of experimentation, no obvious toxicity was observed with the anti-VEGF compounds or DAPT.

### Preparation of retina

Before examination of retinal neovascularization, *fli*-EGFP-Tg zebrafish were killed by a lethal dose of MS-222, followed by decapitation. Fish heads were immersed immediately in 4% PFA, incubated at 4°C overnight and eyes were enucleated. Retinas were isolated from the other tissues and flat-mounted onto glass slides. As for mouse retinas, adult C57/Bl mice were used and eyes were enucleated after exposure to a lethal dose of CO_2_. The isolated mouse retina was immunohistochemically double stained with a rat anti-mouse CD31 and a mouse anti-human α-SMC actin antibody as previously described [44].

### Confocal analysis

Tissue slides were examined under a confocal microscope (Zeiss Confocal LSM510). Mouse retinas were scanned and 5 thin sections (z-thickness: 4–5 µm ) of each sample were assembled into three-dimensional images. Quantitative analysis from at least 12 different tissue sections was performed using the color range tool of Adobe Photoshop CS2 version 9.0.2 software program.

### Statistic analysis

Data is presented as the mean determinants (±SEM). A standard student *t*-test was used for statistical analysis. *p*<0.05 is considered significant; *p*<0.01 is considered highly significant; and *p*<0.001 is considered extremely significant.

## Results

### Retinal vascular networks in adult zebrafish

Development of the retinal vasculature in zebrafish embryos has been described elsewhere [13]. The retinal vascular structure and architecture were revealed using adult *fli-EGFP* transgenic (*fli*-EGFP-Tg) zebrafish [41]. An overview of the retinal vasculature located at inner limiting membrane showed highly organized vascular patterns with the optic artery (OA) located in the center of the optic disc (Fig S1A–F). Approximately 4–9 main vessel branches derived from the optic artery were distributed in each optic disc and they were further divided 2–5 times (grade I–V) before anastomosing with circumferential vein capillaries (CVC). Branch numbers in the grade I optic arteries varied between individual vessels. In contrast, CVCs directly anastomosed to the finest grade of arterial capillaries and drained into the circumferential vein (CV), which encircled the entire optic disc (Fig. S1A–F). Thus, highly organized and double-ended capillary plexuses formed a unique vascular pattern before entering into the CV. Anastomosed arterial-vein capillary plexuses might be susceptible to growth and sprouting in response to angiogenic stimuli. In contrast to zebrafish, the mouse retinal vasculature in the inner membrane of the retina is radially co-distributed with neurons and glia. Optic arteries and veins form a high density of capillary networks in the inner surface of the posterior retina occupying two thirds of the optic disc (Fig. S1G–H).

### Hypoxia-induced retinal neovascularization in adult zebrafish

**Figure 1:**
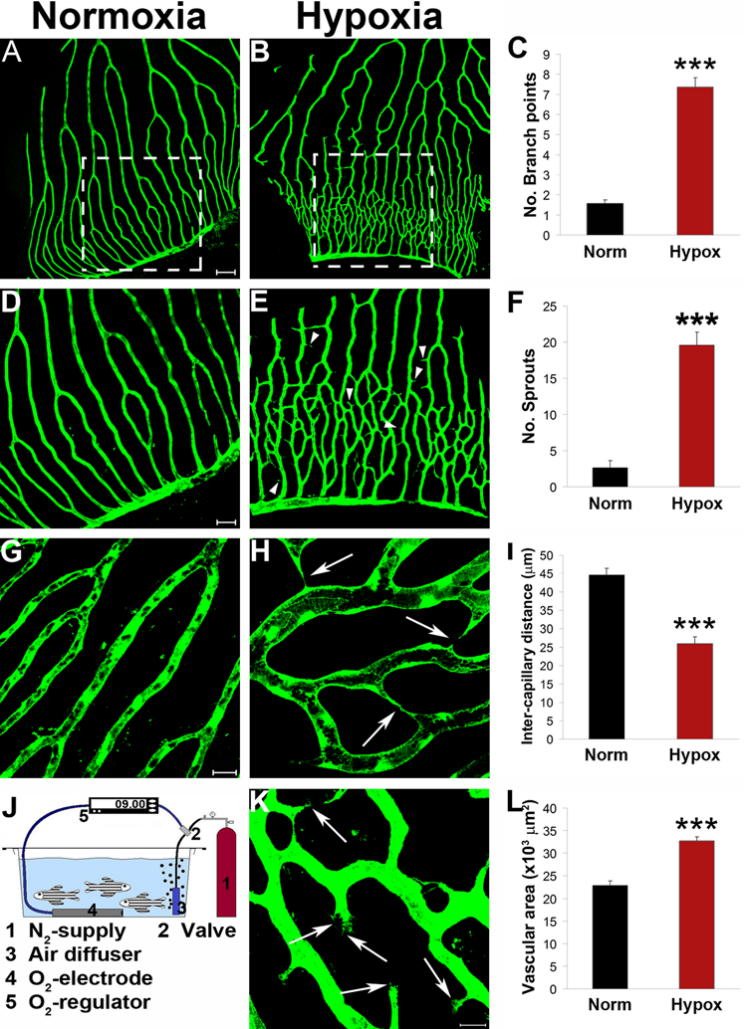
Hypoxia-induced retinal angiogenesis in adult *fli*-EGFP-Tg zebrafish. Adult *fli*-EGFP-Tg zebrafish were placed in a hypoxic aquaria and air saturation in the water was controlled at 10% (820 ppb) by an automated device (J). After 12-days exposure to this hypoxic environment, retinal angiogenesis in the capillary plexuses was detected (B, E, H, and K). Corresponding areas of the retinal vasculture exposed to normoxia were used as controls (A, D and G). Numbers of new vascular branches and sprouts, intercapillary distances, and total vascularization areas were accurately quantified (C, F, I, and L). Yellow arrowheads point to vascular sprouts. Yellow arrows point to endothelial tips. Data represents mean determinants of 11–16 randomized samples. ***p<0.001. Bar in panels A and B = 100 µm; in panels D and E = 50 µm; and in G and H = 20 µm.

To study retinal neovascularization in a pathological settings, we have engineered a hypoxia device for zebrafish (Fig. 1J). In this device, an N_2_ tank was equipped with a pressure indicator, and connected to an air-diffuser submerged in the aquaria water. The aquarium was sealed and the N_2_ flow was regulated by a solenoid valve controlled by an O_2_ regulator. The entire system was automated and O_2_ pressure was self-regulated. Tested drugs could be directed added into water of the sealed aquarium.

Under hypoxia, the optic capillary plexuses of optic arterioles and veins formed new sprouts that during 12 days in 10% air saturation grew to become a high density of capillary networks as compared with those of controls (Fig. 1A, B, D, E, G, H, and K). Interestingly, most capillary sprouts were anastomosed or fused with other sprouts derived from neighboring capillaries (Fig. 1H). In addition, capillary endothelial cell tips were formed at the leading edge of the sprouts (Fig. 1K). Owing to the highly organized vascular architecture of retinal vessels, new vessel branches, sprouts, intercapillary distances, and vascularization area were accurately quantified and significant differences existed between normoxia- and hypoxia-treated zebrafish (Fig. 1C, F, I, and L).

**Figure 2:**
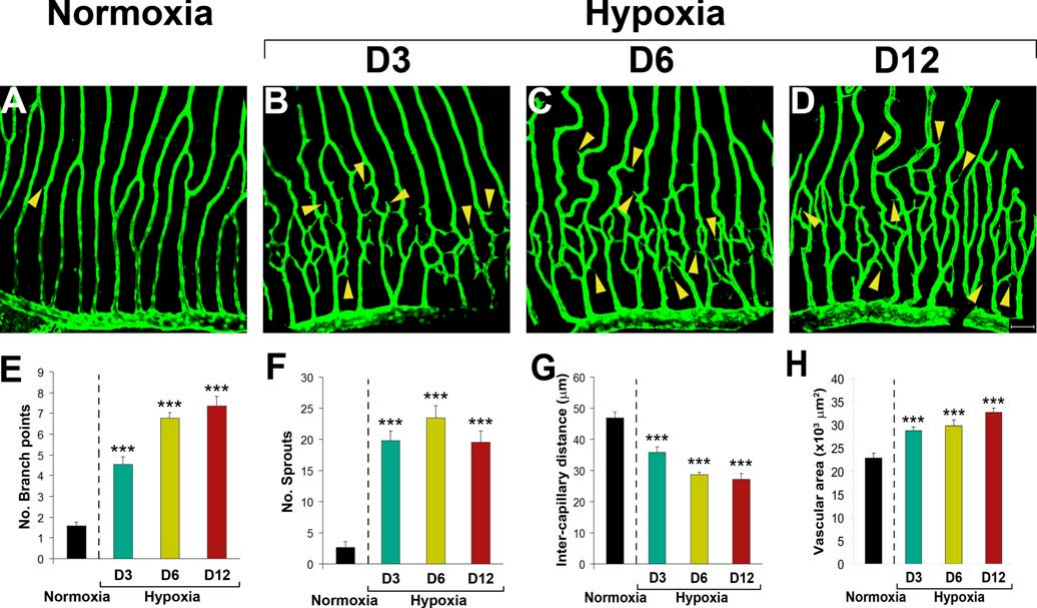
Time-course of hypoxia-induced retinal neovascularization. Hypoxia-induced retinal neovascularization in adult *fli*-EGFP-Tg zebrafish was kinetically monitored (A–H). Angiogenic sprouts were readily formed at day 2 and became overwhelmingly pronounced at day 3 (B) after exposure to hypoxia. The hypoxia-induced retinal angiogenic vessels continued to grow between days 3–12 (B–D). A maximal angiogenic response was detected at day 12. Quantification of new vessel branches (E), sprouts (F) intercapillary distances (G), and total vascularization areas (H) showed significant differences at all time points. Yellow arrowheads point to vascular sprouts. Data represents mean determinants of 11–16 randomized samples. ***p<0.001. Bar = 50 µm.

Time course analysis showed that retinal neovascularization became obvious after exposure to hypoxia for 3 days and increased angiogenic responses were detected at day 6 and day 12 (Fig. 2A–H). At day 12, retinal neovascularization reached a plateau of maximal angiogenic responses. Quantification analysis of the formation of branches and sprouts, intercapillary distances and vascularization areas of the capillary plexuses showed significant differences as compared with those of controls at all time points after exposure to hypoxia, suggesting that retinal vascularization is a relatively rapid process in adult zebrafish.

**Figure 3:**
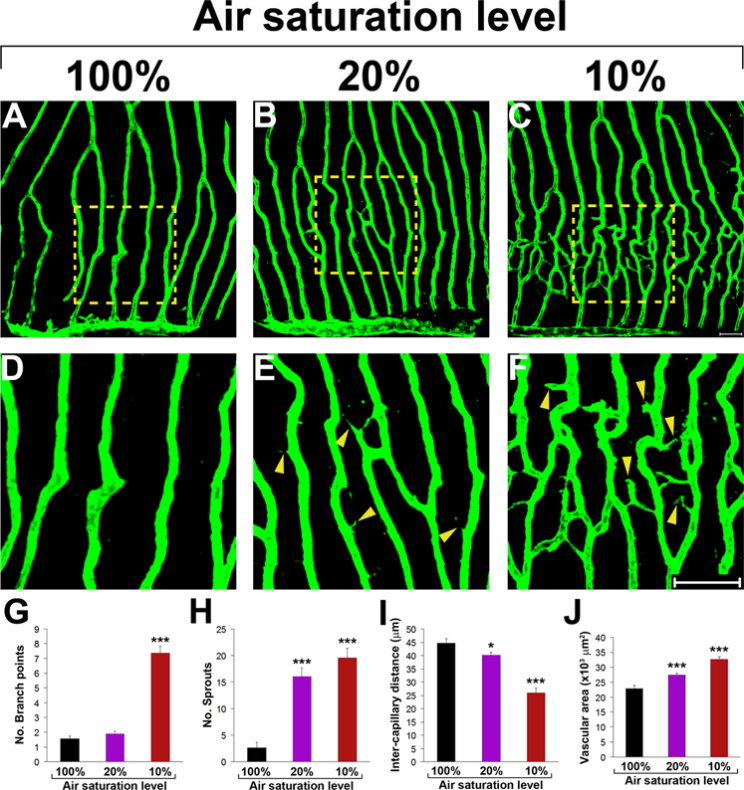
Dose-dependent hypoxia-induced retinal neovascularization. Adult *fli*-EGFP-Tg zebrafish were exposed to 20% or 10% air-saturated water for 6 days. Retinal neovascularization was analyzed using whole-mount confocal analysis and quantified as branching points, numbers of sprouts, intercapillary distances, and total vascularization areas (A–J). Yellow arrowheads point to vascular sprouts. Data represents mean determinants of 11–25 randomized samples. *p<0.05. ***p<0.001. Bar = 50 µm

Exposure of adult zebrafish to various concentrations of air-saturated water showed a dose-dependent angiogenic response to hypoxia (Fig. 3A–J). Although a significant number of angiogenic sprouts occurred at 20% air, the formation of new branches was barely detectable. In contrast, overwhelming retinal angiogenesis was detected in response to 10% air saturation. It should be indicated that a considerable number of adult zebrafish would die if the air saturation was below 10%.

### Blockage of retinal neovascularization by oral anti-VEGF agents

**Figure 4:**
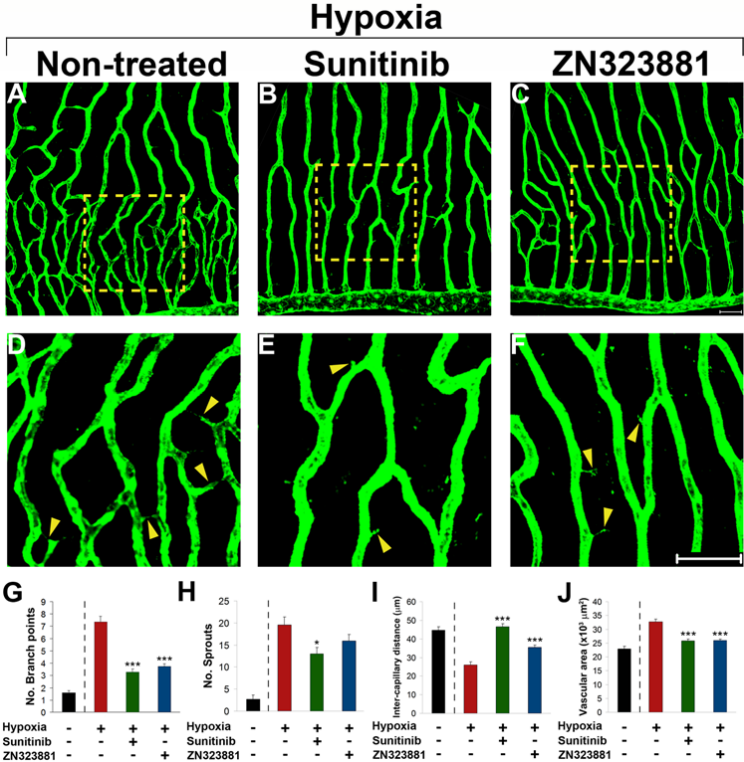
Inhibition of retinal neovascularization by orally active anti-VEGF drugs. Adult *fli*-EGFP-Tg zebrafish were exposed to 10 % hypoxia in the absence (A) or presence of sunitinib (B and E) or ZN323881 (C and F) anti-VEGF small molecules for 14 days. Retinal neovascularization was analyzed using whole-mount confocal analysis and quantified as branching points (G), numbers of sprouts (H), intercapillary distances (I), and total vascularization area (J). Yellow arrowheads point to vascular sprouts. Data represents mean determinants of 11–29 randomized samples. *p<0.05. ***p<0.001. Bar = 50 µm.

It is known that hypoxia induces VEGF expression via the hypoxia inducible transcription factor (HIF) system [18], [42]. To study the role of VEGF and VEGFRs in hypoxia-induced retinal neovascularization in adult zebrafish, known orally active VEGFR inhibitors were tested. Sunitinib and ZN323881, two potent anti-VEGFR-2 agents, almost completely blocked hypoxia-induced retinal neovascularization (Fig. 4A–F). Formation of new vascular branches was virtually totally inhibited by sunitinib and ZN323881 (Fig. 4A–H). Similarly, intercapillary distances and retinal neovascularization areas were also normalized to the levels of retinas exposed to normoxia (Fig. 4I and J). These findings indicate that the hypoxia-triggered VEGF signaling pathway is the primary angiogenic driving force for retinal neovascularization in adult zebrafish. Thus, our zebrafish retinal angiogenesis model recapitulates clinical disease settings showing that VEGF plays a pivotal role in pathological neovascularization in the retina.

### Regulation of hypoxia-induced retinal angiogenesis by the Notch signaling pathway

**Figure 5:**
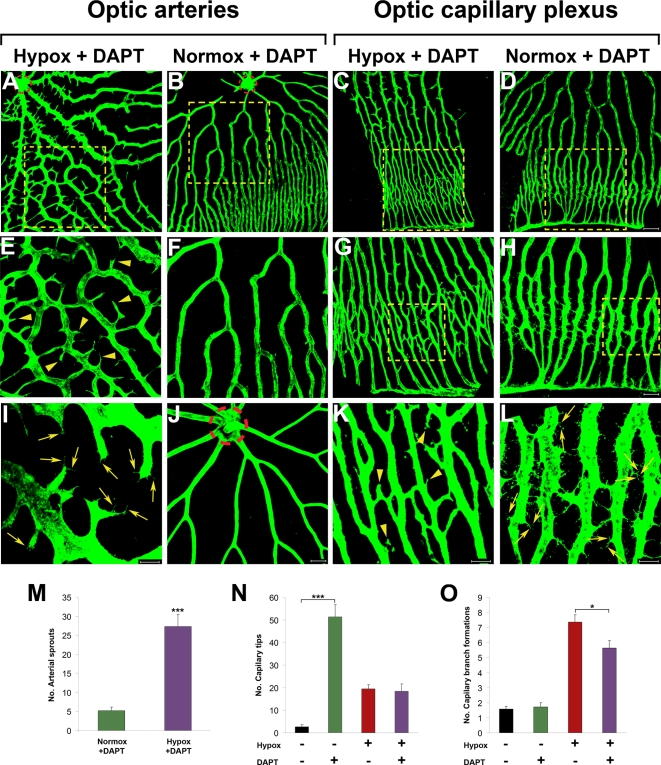
Inhibition of Notch under hypoxia and normoxia. Adult *fli*-EGFP-Tg zebrafish were exposed to 10 µM DAPT under hypoxia for 5 days (A, E, I, C, G, and K) or under normoxia for 6 days (B, D, F, H, and L). Control optic arteries without exposure to hypoxia or DAPT are shown in (J). E–H are amplified images of the inserts of A–D. Retinal neovascularization was analyzed using whole-mount confocal analysis and quantified as numbers of arterial sprouts (M), capillary tips (N) or branch formations (O).Yellow arrowheads point to vascular sprouts. Yellow arrows point to endothelial tips. The dashed red lines encircle the central optic artery. Data represent mean determinants of 11–13 randomized samples. *p<0.05. ***p<0.001. Bars in A–D = 100 µm; Bars in A–D = 50 µm; and Bars in A–D = 20 µm.

The Notch signaling pathway has recently been reported to regulate vascular tip cells formation, vascular sprouting and vascular remodeling [36], [39], [40]. To study molecular mechanisms of hypoxia-induced retinal angiogenesis, the Notch signaling pathway was inhibited by addition of DAPT, a specific inhibitor for γ-secretase, which is essential for notch activation. Interestingly, in the presence of DAPT, virtually all optic arteries form an exceptionally high density of sprouts, which constituted a disorganized vascular network in proximity to the central optical artery (Fig. 5A, E, and I). High magnification analysis of these arterial sprouts revealed endothelial tip cells forming at the migrating leading edges (Fig. 5I, arrows). The arterial sprout/branch formation was completely dependent on tissue hypoxia. Under normoxia, DAPT did not induce the formation of any arterial sprouts/branches, suggesting that hypoxia was the driving force when the Notch signaling was inhibited (Fig. 5B and F).

Interestingly, inhibition of the Notch signaling pathways by DAPT did not increase the hypoxia-induced sprouting and branch formation in the optic capillary plexus area (Fig. 5C, G, and K), suggesting that the negative feedback effect of Notch under tissue hypoxia was limited to the arterial vasculature close to the central optical artery. Surprisingly, inhibition of the Notch under normoxia led to formation of endothelial cell tips in the entire area of capillary plexuses (Fig. 5D, H, and L). These capillary endothelial cell tips were randomly formed from all capillary endothelial cells and were non-directionally distributed in all capillary plexuses (Fig. 5L). Intriguingly, these capillary endothelial tips were rarely detectable under hypoxia in the presence of DAPT, suggesting the hypoxia might suppress endothelial tip cell formation in capillary networks.

## Discussion

Retinopathy, AMD, and other retinal disorders caused by the diabetes epidemic and the change in life style demands development of effective and less expensive targeted therapeutic drugs. For this purpose, development of better animal disease models that recapitulate pathological neovascularization in the retina and clinical retinopathy is urgently needed. Such an in vivo disease model in adult animals would serve as a platform for understanding the underlying mechanism of retinal angiogenesis, for discovery of novel antiangiogenic drugs, and for evaluation of therapeutic efficacy of existing antiangiogenic agents. Unfortunately, a simple and clinically relevant animal model does not exist [5], [6]. In this paper, we report that an adult zebrafish retinal angiogenesis model imitates pathological retinal neovascularization that is relevant to clinical settings . To the best of our knowledge, this is the first report describing the development of a non-invasive adult zebrafish model to study retinal angiogenesis.

A zebrafish retinopathy model is highly relevant to human retinal disorders. Zebrafish and human retinal vasculatures share some common features. Anatomically, the central retinal artery in zebrafish and humans contributes to angiogenesis and the formation capillary plexuses [13]. Developmentally, an initial hyaloid vasculature is associated with the lens and becomes separated at later stage of development [13] (our unpublished data). Similar to humans, the retinal vasculature in the adult zebrafish is also enriched in pericytes and shares some common compositions of ECM and basal lamina [13]. Our zebrafish model has several significant advantages as compared with other existing retinal angiogenesis models including the most commonly used ROP and laser-induced choroidal neovascularization. These include: 1) Non-invasive induction of retinal angiogenesis in the adult animal; 2) Highly reproducible retinal neovascularization in capillary plexuses; 3) Simple, easy and fast experimental procedures; 4) *Fli*-EGFP-Tg zebrafish avoids immunohistochemical procedures; 5) Easy approval by the ethical committee; 6) Low-cost zebrafish facility; 7) Screening of antiangiogenic drugs in a real disease model; 8) Study of vascular patterning, remodeling, and molecular mechanisms of pathological angiogenesis; and 9) Therapeutic evaluation of orally active antiangiogenic agents.

In this adult zebrafish model, we have discovered some unexpected roles of the Notch signaling pathway in relation to tissue hypoxia for regulation of retinal angiogenesis. Under normoxia, inhibition of Notch only leads to capillary endothelial tip sprouting without stimulating the formation of new capillaries. These findings suggest that Notch signaling restricts capillary tip sprouting under physiological conditions. However, without hypoxia as a driving force the formation of capillary endothelial tips would not lead to establishment of functional capillary networks. Inhibition of Notch in the presence of hypoxia completely alters the angiogenic targets from capillary networks to the arterial vasculature. Although the underlying mechanisms remain uncharacterized, it is possible that inhibition of the Notch pathway might lead to repelling of pericytes or smooth muscle cells from arterial vessels, which then become susceptible to VEGF stimulation. This would not explain why capillary tip cells disappeared under hypoxia and Notch inhibition. Probably, hypoxia might inhibit capillary tip cell formation in the absence of notch. These interesting findings warrant further mechanistic investigation. Nevertheless, our findings suggest that hypoxia might alter the target of Notch inhibitors. For development of therapeutic agents targeting the Notch signaling pathway for the treatment of ocular disorders, it is pivotally important to understand the relation between Notch and tissue hypoxia since pathological retinas in most ocular disorders including diabetic retinopathy, AMD and ROP are exposed to severe tissue hypoxia.

Both AMD and diabetic retinopathy are adult-onset disorders characterized by the formation of primitive, leaky and disorganized vascular networks, which grow into the vitreous [4], [17]. These features of the retinal vasculature might reflect the unique aspects of VEGF function that include induction of disorganized, highly leaky, tortuous and primitive vascular networks. A large body of studies demonstrates that VEGF is the primary molecule responsible for pathological angiogenesis in the retina of AMD and diabetic retinopathy [16], [17], [19]–[21]. Indeed, clinical experiences with anti-VEGF agents for the treatment of AMD have shown remarkably beneficial effects [16]. Expression levels of VEGF are primarily regulated by tissue hypoxia *via* the HIF system [18], [42]. With no exception in the pathological retina of AMD and retinopathy, hypoxia is the key element of retinal neovascularization. However in mammalian models, it is difficult to develop a hypoxia-induced retinal angiogenesis model in adult animals. Although the infant ROP model in mice is also based on mechanisms of the hypoxia-induced angiogenesis, exposure to normal air is considered as a relative hypoxia. Thus, this model is not considered as a true disease model.

Our findings show that similar to humans the retina of zebrafish responds to hypoxia, which initiates pathological angiogenesis. We further demonstrate that orally active angiogenesis inhibitors could significantly inhibit retinal neovascularization, suggesting that VEGF is responsible for the hypoxia-induced angiogenesis.

## Supporting Information

Figure S1 (0.25 MB PDF)
