# Supplementary material for: Hypoxia-Induced Retinal Angiogenesis in Zebrafish as a Model to Study Retinopathy
Source: PLoS One. 2008 Jul 23;3(7):e2748. doi: 10.1371/journal.pone.0002748 (PMC2447178; doi:10.1371/journal.pone.0002748)
Supplement: Figure S1 — (0.25 MB PDF) [file pone.0002748.s001.pdf]

Supplementary information

Fig. S1 Comparison of the retinal vasculature in zebrafish and mice. Dorsal (a) and lateral views (c and e) of the retinal vasculature in zebrafish. The architecture and patterning of the retinal vasculature are highly organized with the optic artery (OA) located in the center of the optic disc (a-f, OA). Approximately 4-9 branches (Grade I) are derived from the OA and they further divide 2-5 times (Grade II-V) before the formation of capillary plexuses, which anastomose with vein capillaries that are collected in the circumferential vein (a-f, CV). The mouse retinal vasculature contains high density large and microvascular networks derived from the central retinal artery (CRA) and central retinal vein (CRV) (g and h). Red=CD31 positive signal; green=α-SMA positive signal; and yellow=double positive signal.

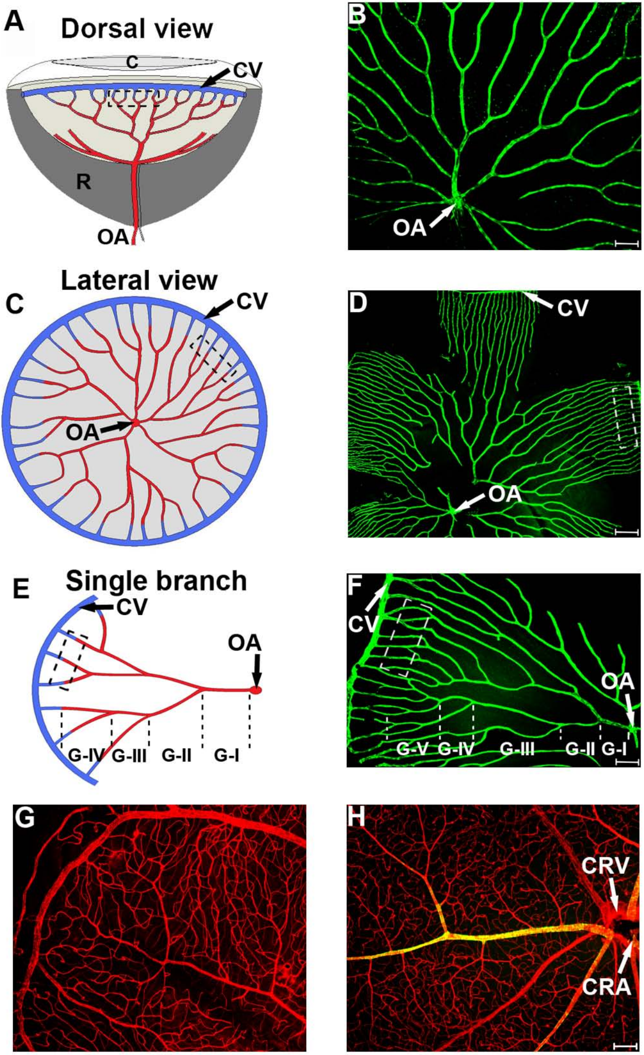
A
Dorsal view
C
CV
R
OA
B
OA
Lateral view
C
CV
OA
D
CV
OA
E
Single branch
CV
OA
G-IV
G-III
G-II
G-I
F
CV
OA
G-V
G-IV
G-III
G-II
G-I
G
H
CRV
CRA
